# Recombinant Flag-tagged E1E2 glycoproteins from three hepatitis C virus genotypes are biologically functional and elicit cross-reactive neutralizing antibodies in mice

**DOI:** 10.1016/j.virol.2018.03.026

**Published:** 2018-06

**Authors:** Vasil B. Krapchev, Malgorzata Rychłowska, Alicja Chmielewska, Karolina Zimmer, Arvind H. Patel, Krystyna Bieńkowska-Szewczyk

**Affiliations:** a Laboratory of Virus Molecular Biology, Intercollegiate Faculty of Biotechnology of UG and MUG, University of Gdansk, 58 Abrahama str., 80-307 Gdansk, Poland; b MRC-University of Glasgow Centre for Virus Research, Sir Michael Stoker Building, Garscube Campus, 464 Bearsden Road, Glasgow G61 1QH, Scotland (UK)

**Keywords:** Hepatitis C virus, E1E2, Envelope glycoproteins, Flag tag, Neutralization, Vaccine

## Abstract

Hepatitis C virus (HCV) is a globally disseminated human pathogen for which no vaccine is currently available. HCV is highly diverse genetically and can be classified into 7 genotypes and multiple sub-types. Due to this antigenic variation, the induction of cross-reactive and at the same time neutralizing antibodies is a challenge in vaccine production. Here we report the analysis of immunogenicity of recombinant HCV envelope glycoproteins from genotypes 1a, 1b and 2a, with a Flag tag inserted in the hypervariable region 1 of E2. This modification did not affect protein expression or conformation or its capacity to bind the crucial virus entry factor, CD81. Importantly, in immunogenicity studies on mice, the purified E2-Flag mutants elicited high-titer, cross-reactive antibodies that were able to neutralize HCV infectious particles from two genotypes tested (1a and 2a). These findings indicate that E1E2-Flag envelope glycoproteins could be important immunogen candidates for vaccine aiming to induce broad HCV-neutralizing responses.

## 1 Introduction

Hepatitis C virus (HCV), a member of the *Flaviviridae* family, is a globally disseminated human pathogen causing liver disease, such as cirrhosis and hepatocellular carcinoma (Alter and Seeff, 2000). Globally, in 2015, an estimated 71 million people were living with chronic HCV infection (WHO, 2017). Despite the recent development of highly effective direct-acting antivirals (DAA) (González-Grande et al., 2016), the infection remains a major health problem worldwide. This is due to the limited availability and high cost of new therapies, low infection awareness and high probability of reinfection in high-risk groups (Baumert et al., 2014). Therefore, an effective prophylactic and/or therapeutic vaccine is still needed to control the virus globally. One of the major obstacles for vaccine development is the extreme genetic variability of HCV, driven by its escape from immune pressure.

The HCV envelope glycoproteins E1 and E2 play a crucial role in the complex process of virus entry into the host cell. They are a primary target for the antiviral adaptive immune response and therefore are important immunogen candidates for the design and development of vaccines against HCV (Wang et al., 2011). The current knowledge of E1E2 structure and functions comes from numerous biochemical, molecular and immunological studies and was recently improved by obtaining the crystal structure of E2 core (Khan et al., 2014, Kong et al., 2013). However, the genetic diversity and the complex structure of the heterodimer formed by E1 and E2 makes them a very difficult research target.

Here we show the construction, purification and broad functional and immunological evaluation of E1E2-based antigens derived from three different HCV genotypes. The E1E2 recombinant proteins were tagged with the Flag tag, for the facilitation of protein isolation and purification. Numerous recombinant Flag-tagged viral proteins have been previously described and efficiently purified by various groups. These include the gp120 of simian immunodeficiency virus (SIV) (Laird and Desrosiers, 2007), ORF virus envelope proteins (Tan et al., 2009) and the VP1 protein from foot-and-mouth disease virus (FMDV) (Lawrence et al., 2013). Furthermore, the Flag tag has been successfully used in the study of HCV for the purification of cell cultured viral particles (HCVcc) (Merz et al., 2011, Prentoe and Bukh, 2011).

We previously identified a site within the hypervariable region 1 (HVR-1) of the genotype 1a HCV strain H77 glycoprotein E2 where a small insertion of 5–6 amino acids was tolerated without a negative effect on the protein structure and function (Rychlowska et al., 2011). Based on that data, in the present report we constructed and analyzed three E1E2 mutants with the Flag octapeptide inserted at amino acid position 409 in the HVR-1 of E2. We show that such an insertion is well tolerated in three different HCV genotypes (1a, 1b and 2a). We also demonstrate that Flag insertion in this site does not hinder protein expression, proper conformation of E2 and the activity of the glycoprotein – E1E2 dimer formation and CD81 binding. Moreover, we examined the immunogenic properties of E1E2-Flag and found that immunization of mice with affinity purified recombinant Flag-tagged proteins induced anti-E2 antibodies capable of neutralizing cell cultured HCV (HCVcc). These results establish the E1E2-Flag as potential vaccine immunogens as well as tools for molecular and antigenic studies.

## 2 Results

### 2.1 Construction and expression of E1E2-Flag glycoproteins

**Fig. 1:**
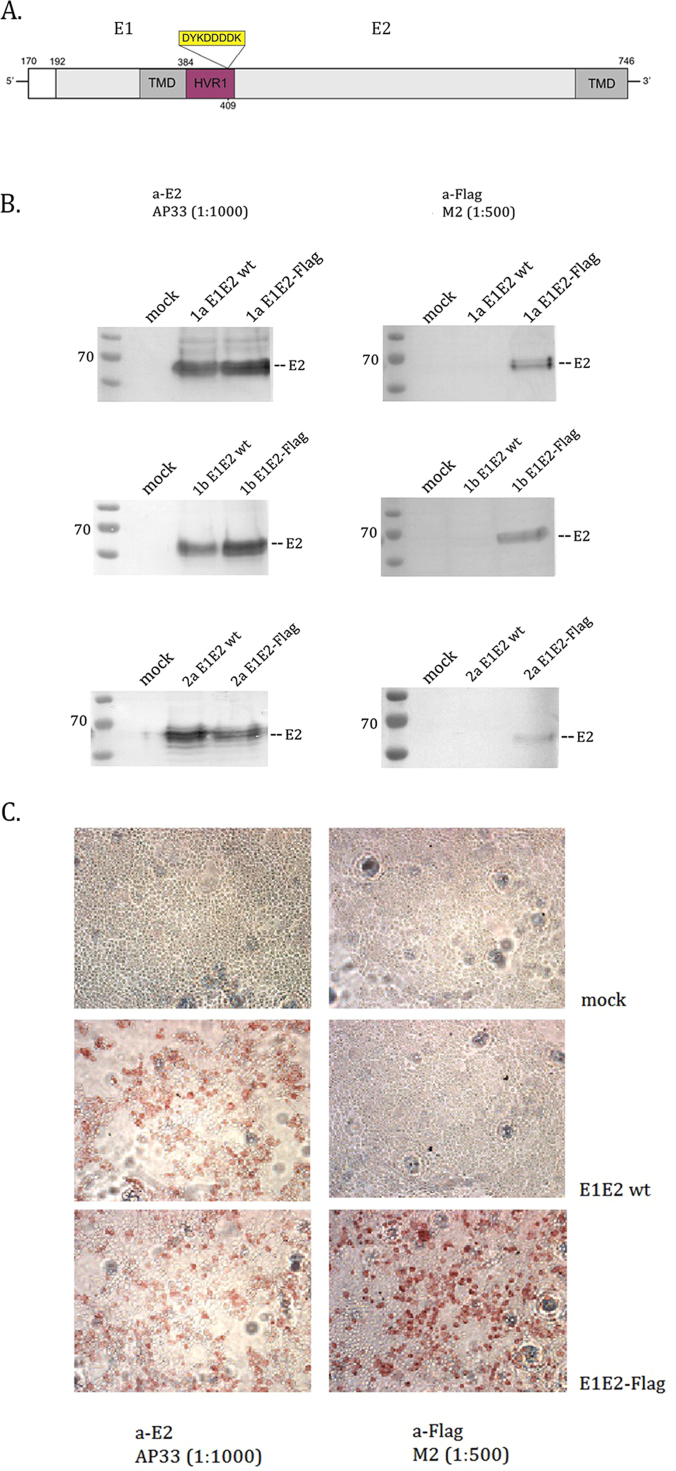
Construction and analysis of the expression of E1E2-Flag glycoproteins. A. Schematic diagram of E1E2-Flag. The numbering corresponds to the amino acid positions in the polyprotein of the genotype 1a H77 strain. TMD – trans-membrane domain; HVR-1 – hypervariable region 1. B. Western blot detection of E2 wt and E2–Flag protein expression in 293T cells 24 h after transfection. Detection using anti-E2 AP33 (left column) or anti-Flag M2 (right column) mAbs, followed by anti-mouse IgG AP conjugate and NBT/BCIP substrates. Mock-transfected 293T lysates were used as a negative control. Loaded amounts were normalized for total protein content. C. Detection of gt1a E2 wt and E2-Flag proteins by immunohistochemical staining in fixed 293T cells. 48 hrs post transfection with either E1E2 wt or E1E2–Flag expression plasmids, the cells were fixed and probed with anti-E2 AP33 or anti-Flag M2 mAbs. Mock transfected HEK cells were used as a negative control.

In this study, we have constructed Flag-tag modified E2 glycoproteins derived from the two HCV genotypes most prevalent in Europe and North America – 1a and 1b (Petruzziello et al., 2016), as well as from genotype 2a, from which the first clone replicating efficiently in cell culture was isolated (Wakita et al., 2005, Zhong et al., 2005, Kato et al., 2006) (Fig. 1. A.). The sequences used for this study were previously described by (Tarr et al., 2007), who amplified E1E2 from patient-derived sera and cloned them into the pcDNA3 expression vector, under the control of the human cytomegalovirus (CMV) promoter. The Flag DYKDDDDK octapeptide tag was introduced at position 409 in E2, immediately preceding the conserved Q410 glutamine residue.

The E1E2-Flag proteins were transiently expressed in 293T cells and the expression was examined by Western blotting and in situ immunostaining. The recombinant proteins were recognized by both the anti-E2 mAb AP33, as well as by the anti-Flag mAb M2 (Fig. 1. B.). E2 proteins were of expected molecular weight of 62 kDa and the expression level was similar to the wild type E2, indicating that the addition of the tag did not affect the efficiency of protein synthesis. Immunohistochemical staining of 293T cells after transfection was performed in parallel to Western blotting in order to visualize the expression of the E2 proteins directly in cells. The detection of immunostained E1E2 wt and Flag from genotype 1a is shown on Fig. 1. C. Similarly, when using an anti-E2 mAb positive signal was observed in cells expressing both unmodified and tagged E1E2, while anti-Flag mAb recognized exclusively the Flag tagged proteins and these results were consistent for all three genotypes. Efficient cell immunostaining with anti-Flag mAb suggested that the Flag epitope was exposed on the surface of recombinant HCV E2 glycoproteins and could be used for further purification purposes.

### 2.2 Affinity purification of E1E2-Flag heterodimers

It has been previously described that HCV E1 and E2 proteins assemble into non-covalent heterodimer (Castelli et al., 2017, Dubuisson et al., 1994, Freedman et al., 2017). Ideally, the insertion of an affinity tag in either E1 or E2 should not interfere in this process and should allow the efficient purification of functional E1E2 heterodimer with correct conformation.

Agarose coupled to the M2 anti-Flag antibody was used as affinity resin to purify E1E2-Flag heterodimers from the lysates of transfected 293T cells. Proteins were expressed transiently for 48 h and purified from cell lysates. To optimize the elution process, two different methods were used – a competition with the 3x Flag peptide and a low pH buffer elution. We observed significantly higher efficiency of E1E2-Flag elution using glycine buffer pH 2.7, which together with its cost effectiveness was the reason why this method was chosen for further experiments. In the optimized elution procedure, the low pH in the eluted fractions was immediately neutralized by Tris-HCl buffer pH 8.8. Consistently with other reports (Meertens et al., 2006, Tscherne et al., 2006), we have observed that short incubation in low pH followed by a return to neutral pH did not result in precipitation or denaturation of HCV glycoproteins. Following analysis of protein content and concentration, selected elution fractions were pooled and concentrated 10x by ultrafiltration.

**Fig. 2:**
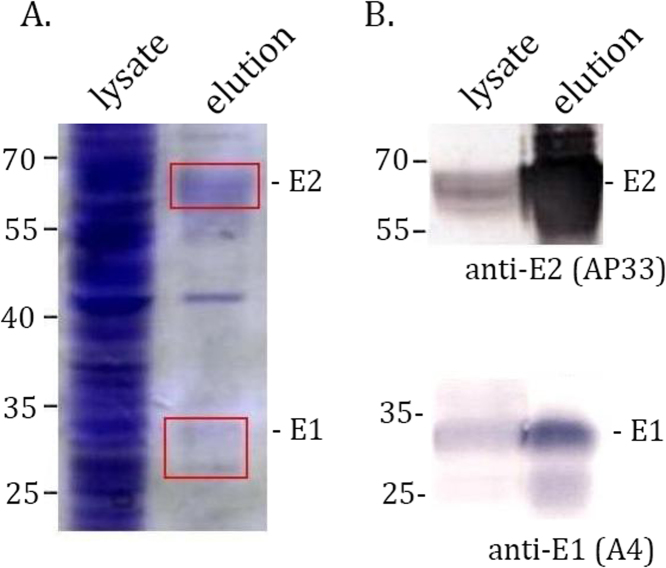
Purification of gt1a E1E2-Flag from transfected 293T cells. 48 hrs post transfection with gt1a E1E2-Flag expression plasmid, the cells were lysed and incubated with the M2 anti-Flag agarose. The eluted fraction was analyzed by A. Coomassie-stained SDS-PAGE (the E2 and E1 bands are indicated by red frames; a ~ 50 kDa band of the H chain of the anti-Flag antibody is visible as the only impurity) and by B. Western blot using the anti-E2 AP33 and the anti-E1 A4 mAbs.

We have estimated the purification efficiency of E1E2-Flag after transfection of 293T cells as 15 µg from 5 × 10^6^ cells growing in stationary cultures. Protein concentration was estimated by Bradford assay and the purity was verified on a Coomassie-stained 10% SDS-PAGE gel and Western blotting. The same purification procedure was used for all three genotypes and an example for the purification of gt1a E1E2-Flag is shown on Fig. 2.

### 2.3 Analysis of E1E2 heterodimer formation

**Fig. 3:**
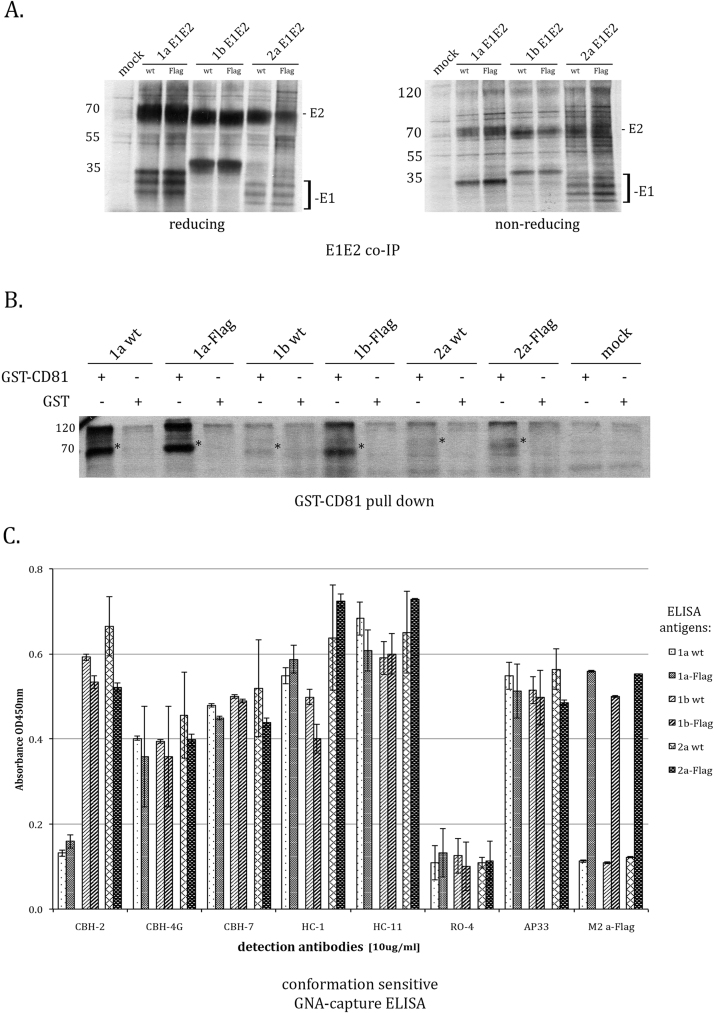
Functional and structural analyses of E1E2-Flag proteins. A. E1 glycoprotein co-immunoprecipitates with E2. 293T cells transiently expressing wt and E1E2-Flag were metabolically radiolabeled with [^35^S]-Met and lysed 48 hrs post transfection. The proteins were immunoprecipitated with the anti-E2 AP33 mAb, separated on 12% SDS-PAGE (under reducing and non-reducing conditions) and detected by radiography. B. Binding of E1E2-Flag to CD81 tested by pull-down assay. GST-hCD81-LEL was bound to glutathione agarose, the batch was divided into aliquot parts and incubated with lysates of 293T cell expressing HCV E1E2. GST bound to glutathione agarose was used as a negative control. The samples were resolved on 12% SDS-PAGE gel and detected with AP33 mAb. Asterisks indicate the position of E2. C. Analysis of conformational epitopes of HCV E1E2-Flag. Lectin-bound E1E2 complexes from transfected 293T cell lysates were probed with a panel of conformation-sensitive anti-E2 antibodies. An irrelevant isotype control hmAb RO4 and the slightly conformation-sensitive anti-E2 mAb AP33 were used as negative and positive controls, respectively. Bars represent the mean values obtained from triplicate experiments; standard deviation of the readings is indicated.

As mentioned before, correctly folded and functional intracellular E1 and E2 proteins form a non-covalent heterodimer. In order to investigate the ability of E2-Flag to bind its partner glycoprotein E1, radioimmunoprecipitation assay (RIPA) was performed. The anti-E2 mAb AP33 was used to precipitate the putative complexes from the lysates of transfected 293T cells. To differentiate between precipitated covalently bound protein aggregates and co-precipitated non-covalent E1E2 heterodimers the proteins were analyzed by SDS-PAGE under reducing and non-reducing conditions (Fig. 3. A.). As seen on the non-reducing gel, monomeric E1 and E2 can be detected apart from a higher molecular weight, b-mercaptoethanol-resistant multimers. Such monomeric forms indicate that at least a part of E1 and E2-Flag are able to associate into functional non-covalent heterodimers. For glycoprotein E1 multiple bands could be detected, most probably representing different glycosylation states of the protein, as observed by others in earlier studies (Dubuisson et al., 2000). These results were further confirmed using anti-Flag antibody for IP. As expected, the M2 mAb co-precipitated E1 together with E2 from the Flag mutants exclusively (data not shown). Concluding, insertion of the Flag epitope did not influence the heterodimerisation process of E1 and E2 HCV glycoproteins expressed transiently in HEK 293T cells.

### 2.4 E1E2-Flag proteins bind to HCV receptor CD81

As a next step, we investigated the ability of the E1E2-Flag proteins to bind the HCV receptor CD81. CD81 is one of the essential entry receptors and blocking E1E2 interaction with CD81 neutralizes HCV infection in vitro (Fénéant et al., 2014). The regions in E2 and CD81 responsible for the interaction have been mapped (Drummer et al., 2006) and CD81 binding is often used as a functional “quality control” assay for recombinant HCV glycoproteins (Heile et al., 2000). Here, the assay was based on purified recombinant large extracellular loop (LEL) of human CD81 protein, fused to GST. Immobilized GST-hCD81-LEL was used as bait in pull-down assay to bind E1E2 proteins from cell lysates (Fig. 3. B.). As a control, GST alone was used as bait. Recombinant CD81, but not GST alone, was able to pull down detectable amounts of all the HCV E1E2 variants but with different efficiency. Results indicate that the addition of the Flag epitope did not disrupt the CD81 binding site on E2.

### 2.5 The addition of the Flag epitope does not affect the folding and conformational epitopes of glycoprotein E2

To analyze the influence of the insertion of Flag epitope in the HVR-1 region on E2 folding, we used a panel of human monoclonal antibodies recognizing different conformational epitopes in E2. First, we immobilized the recombinant E1E2 onto ELISA plates by GNA-capture. The lectin bound proteins were then probed with conformation-sensitive antibodies mapped to different domains in HCV E2. The load of E1E2 proteins immobilized on the ELISA plates was normalized based on the reactivity with the higly cross-reactive, slightly conformation-sensitive (Potter et al., 2012) anti-E2 AP33 and anti-Flag mAbs (Fig. 3. C.).

We did not observe a decreased binding of conformation sensitive mAbs to the Flag modified E2. All tested mAbs bound Flag tag mutants with affinity similar to that observed for wild type proteins, indicating correct conformation of E1E2-Flag recombinant proteins. Consistent with previously published report by (Owsianka et al., 2008), one of the conformation sensitive antibodies, namely CBH-2, failed to recognize either the wt or the Flag-modified E2 from HCV genotype 1a (H77c). However, all other antibodies used in this assay readily detected our recombinant proteins.

### 2.6 The E1E2-Flag antigens are immunogenic in mice and induce cross-reactive polyclonal antibodies

**Fig. 4:**
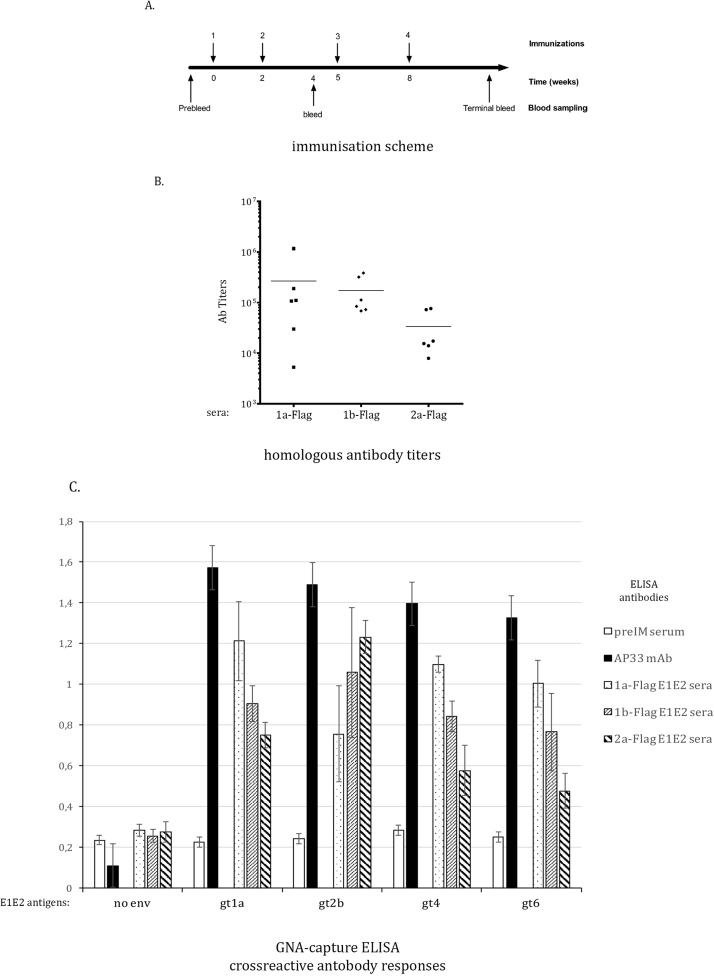
Immunological analyses of E1E2-Flag mouse sera, A. Immunization scheme. C57BL/6 mice were primed at day 0 with full length E1E2-Flag (gt1a, 1b or 2a), and boosted with the same antigen at weeks 2, 5 and 8. The terminal bleed was at week 10. B. Quantification of humoral responses induced by vaccination with E1E2-Flag proteins. Lectin-bound gt1a, 1b or 2a E1E2-Flag immunogens were probed with the corresponding homologous E1E2-Flag sera in three-step serial dilutions (starting from 1:500, up to 1:10^6^). Absorbance was measured at 450 nm. Each dot on the plot represents one animal (■ - 1a-Flag, ♦ - 1b-Flag, • - 2a-Flag). The antibody titer was estimated as the serum concentration, at which binding was 2 times higher than the pre-immune (preIM) control. The horizontal bars represent the median value for each group. C. Cross-reactivity of mice sera against selected HCV genotypes. Lectin-bound E1E2 complexes from 293T cell lysates, transfected with gt1a (the heterologous strain UKN 1a.12.18), gt2b (UKN 2b1.1), gt4 (UKN 4.21.16) or gt6 (UKN 6.5.34) were probed with each of the E1E2-Flag sera, followed by anti-mouse IgG–HRP conjugate and TMB substrate. Absorbance was measured at 450 nm. The anti-E2 AP33 mAb was used as a positive and the anti-Flag M2 and preimmune (preIM) serum as negative controls, respectively. Mock transfected 293T cell lysates (no env) were used for the estimation of background signal. Error bars represent standard deviations of the individual mouse responses.

The purified E1E2-Flag glycoproteins were used as antigens for mouse immunization. Three groups of six female, 5–6 weeks of age pathogen-free C57BL/6 mice per group were intraperitoneally injected with 5 μg E1E2-Flag per animal as shown on Fig. 4. A.

The total blood was collected from the heart of each animal (terminal bleed) and the serum was isolated by centrifugation. Few of the animals had fluid retention in the abdomen, which was also collected. The polyclonal sera were analyzed for their ability to recognize the immunizing antigens as well as for their cross-reactivity against antigens from different HCV genotypes.

First, the humoral responses induced by vaccination with E1E2-Flag proteins were quantified. The relative antibody titer of each serum was estimated by GNA-capture ELISA against the homologous immunizing antigen (Fig. 4. B.). All tested immunogens were able to induce high antibody responses in the vaccinated animals. We detected higher median titers for genotype 1a (10.9 × 10^4^) and gt1b (9.84 × 10^4^) E1E2 immunogens, in comparison to the gt2a (1.64 × 10^4^) E1E2 immunogen (p = 0.01).

Next, we examined the cross-reactivity of the induced antibodies against E1E2 glycoproteins derived from heterologous HCV genotypes. The obtaining of humoral cross-reactivity is a challenging task, due to the high genetic diversity of HCV. The GNA-capture ELISA experiments indicated that the immunizations induced both homologous and heterologous antibody responses. The GNA-capture ELISA experiments, in which E1E2 glycoproteins from different genotypes were used as antigens, confirmed that the analyzed sera successfully recognized HCV genotypes 1a (UKN 1a.12.18), 2b (UKN 2b1.1), 4 (UKN 4.21.16) and 6 (UKN 6.5.34) (Fig. 4. C.). We observed induction of broad and strongly cross-reactive antibody responses in case of gt1a E1E2-Flag antigen and lower in case of gt1b. Antibodies induced by the gt2a E1E2-Flag immunogen had lowest cross-reactivity; in particular against E1E2 complexes from genotypes 4 and 6, while homologous antigen binding was high.

### 2.7 The E1E2-Flag polyclonal mouse sera neutralize HCV entry in vitro

The sera were furthermore tested for their ability to block the entry of cell-cultured hepatitis C virus (HCVcc) into hepatocytes by in vitro neutralization assay. To assess neutralizing activity, pre-immune (preIM) and terminal bleed sera were tested against gt1a (H77/JFH1 chimera) and 2a (JFH1) cell-cultured HCV viruses. 50 focus-forming units (FFU) of H77/JFH1/HQL or JFH-AM71 – cell-culture adapted, high-titer producing viruses (data not shown), were pre-incubated with the appropriate inhibitory serum or control antibody prior to infection. For the accurate quantitation of HCV replication in cell culture, we used the cell-based secreted alkaline phosphatase (SEAP) reporter assay. This assay is based on the Huh7-J20 reporter cell line, which stably expresses the fusion protein enhanced green fluorescent protein (EGFP) fused in-frame to the SEAP via a recognition sequence of the viral NS3/4A serine protease. Upon virus infection, SEAP is cleaved by the HCV NS3/4A protease leading to its release into the culture medium (Iro et al., 2009).

**Fig. 5:**
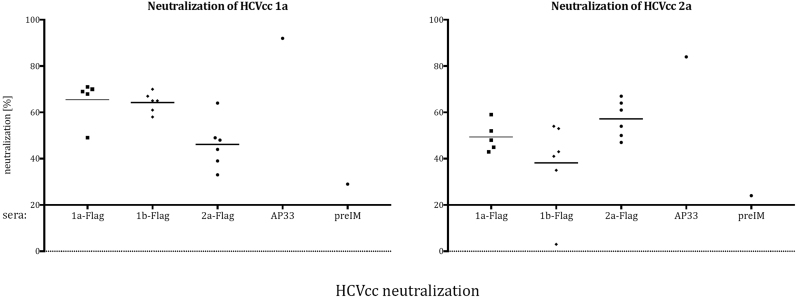
Neutralization of HCVcc by the E1E2-Flag polyclonal mouse sera. Huh-7-J20 cells were infected with 50 FFU of virus, pre-incubated with the appropriate inhibitory or control antibody (H77/JFH1/HQL chimera – left panel; JFH1-AM71 – right panel). After 48 hrs, the infectivity was determined by measurement of the SEAP activity released into the medium. Each dot on the plot represents one animal (■ - 1a-Flag, ♦ - 1b-Flag, • - 2a-Flag). The anti-E2 AP33 mAb and preIM serum were used as positive and negative controls, respectively. The horizontal bars represent the median value for each group.

As expected, the 1a-Flag and 1b-Flag sera neutralized the 1a HCVcc chimeric virus more efficiently – with median values of 66 and 64 per cent respectively, versus the 2a-Flag with a median value of 47% neutralization (Fig. 5). The contrary was observed for the JFH1 virus, which was neutralized stronger by the 2a-Flag sera (median of 58%) and less efficiently by 1a- (50%) and 1b-Flag (38%), with p values of 0.02 and 0.01, respectively. The median value for gt1b was decreased by one individual mouse serum, which presented no neutralization activity. In comparison, the anti-E2 mAb AP33 neutralized HCVcc by over 80% and the preIM serum had a 22–25% unspecific blocking of entry.

## 3 Discussion

In the present study, three Flag-tagged mutants of HCV E1E2 from genotypes 1a, 1b and 2a were generated, with the in-frame Flag insertion at position 409 in the HVR-1 of E2. We have previously shown (Rychlowska et al., 2011) that this place tolerates small insertions without major defects in E2 from gt1a (strain H). Here we confirm this phenomenon is not restricted to the H77 clone alone.

The insertion site was different than in other reports where Flag insertions were mostly replacing HVR-1 (Cocquerel et al., 2003, Takahashi et al., 2010) or added to the N-terminus of E2 via variety of linkers (Merz et al., 2011, Prentoe and Bukh, 2011). The choice of Flag placement is essential for the preservation of the E2 regions crucial for virus entry to host cells and for E2 immunogenicity (Vasiliauskaite et al., 2017). The E1E2-Flag proteins were efficiently expressed as shown by Western blotting and in situ immunostaining of E1E2 wt and Flag expressed in 293T cells. Finally, we standardized the procedure for production and purification of E1E2-Flag proteins. Using affinity-based chromatography Flag-tagged E1E2 were purified from cellular protein contaminants by over a 1000-fold as measured by protein determinations of the purification fractions. Using the M2 resin, around 30% of the input of bound protein could be eluted in batch purification.

Furthermore, we could show the Flag mutants retained their authentic biological properties, as E2 was able to form a heterodimer with E1. Moreover, the recombinant E1E2 were able to bind to the cellular receptor CD81, which is a crucial factor in the HCV life cycle. However, as reported earlier by (Roccasecca et al., 2003), a substantial difference in binding efficiency between the three genotypes was observed, with proteins derived from gt1b and 2a showing significantly lower binding than the gt1a protein. Interestingly, our 1b and 2a Flag mutants bound CD81 stronger than their homologous wild types, indicating a possible conformational modification in E2, leading to a better presentation of the region immediately succeeding the HVR-1.

A panel of human monoclonal conformation-sensitive Abs (a kind gift from S. K. Foung), previously described by (Hadlock et al., 2000) and (Owsianka et al., 2008) was used for the analysis of correct folding and conformational epitopes of the recombinant E1E2. These mAbs recognize different parts of the E2 structure and are phylogenetically grouped in three groups, each binding to a different domain in E2 (Keck et al., 2004). The CBH-4 G, CBH-7, HC-1 and HC-11 mAbs recognized the Flagged E1E2 from all three genotypes with similar affinity as compared to the wt. The CBH-2 mAb, recognized gt1b and 2a but not 1a, which is consistent with previous report (Owsianka et al., 2008), where this antibody failed to bind to the H77 clone.

The previous studies used the Flag tag for purification of viral particles of cell-cultured HCV (HCVcc), however no immunological studies were performed. We used purified gt1a, 1b and 2a E1E2-Flag proteins for mouse immunizations and they elicited strong immune responses, raising high titers of antibodies after introducing as little as 5 µg protein per animal. In GNA-capture ELISA, the mouse polyclonal sera reacted with the E1E2-Flag immunogen as well as to the homologous wild type. The median antibody titer values were as high as 10.9 × 10^4^ for gt1a-Flag and 9.84 × 10^4^ for gt1b, while in the 2a-Flag group were tenfold lower (1.64 × 10^4^).

Due to the genetic diversity of HCV it was important to check the cross-genotypic reactivity of the sera. In GNA-capture ELISA, we could show that 1a- and 1b-Flag recognize best E1E2 from gt4 (UKN 4.21.16) and 6 (UKN 6.5.34), while for gt2b (UKN 2b1.1) the strongest reaction was detected using gt2a-Flag. Overall, the best cross-reactivity was observed after immunizing with E1E2-Flag from genotype 1a. In Western blot under reducing conditions, reactions were either very weak or absent (data not shown), which when compared to the results from the ELISA experiments suggests the presence of conformationally sensitive antibodies in the sera.

It has been shown that spontaneous resolution of HCV infection is associated with potent and long-lasting T-cell responses. Moreover, early appearance of broadly neutralizing antibody responses leads to successful clearance of infection (Osburn et al., 2014, Pestka et al., 2007). However, recent approaches to induce virus-specific CD8 T-cells with vectored vaccines encoding non-structural HCV proteins failed to suppress viral load in chronically infected humans (Kelly et al., 2016) and chimpanzees (Callendret et al., 2014). In fact, meta-analysis study of HCV experimental vaccinations in chimpanzees demonstrated that vaccine formulations containing structural proteins, were associated with more significant protective immune responses than vaccines based solely on non-structural proteins (Dahari et al., 2010). To date, HCV structural E1 and E2 proteins have been tested as immunogens in variety of modalities including virus-like particles (Czarnota et al., 2016, Garrone et al., 2011), recombinant proteins (Freedman et al., 2017, Grzyb et al., 2016, Logan et al., 2017) and prime-boost regimens with the use of viral vectors (Chmielewska et al., 2014, Reyes-del Valle et al., 2012). These different strategies were able to raise high-titer cross-neutralizing antibody responses in animals. The most advanced trial based on E1E2 complex purified from CHO cells induced broadly cross-neutralizing antibodies in animals and humans in phase I clinical trials (Wong et al., 2014).

Finally, we investigated the neutralization potential of the polyclonal sera. Most mice sera inhibited HCVcc viral entry by 45–65% for the gt1a H77/JFH chimera and 38–58% for the gt2a JFH1 virus. Recently, vaccines eliciting neutralizing antibodies to structural epitopes have been evaluated as protective against HIV (Jardine et al., 2013), respiratory syncytial virus (RSV) (Correia et al., 2014) and influenza (Mallajosyula et al., 2014, Yassine et al., 2015). This further supports the notion that a universal vaccine against HCV should include components inducing strong humoral responses to conserved structural epitopes and encourages the search for ideal E1E2 immunogen modalities.

The E1E2-Flag mutants reported here could turn out to be important candidates for vaccine antigens. Furthermore, they can be used as authentic counterparts of native proteins in structural studies, diagnostic tests or for the generation of new potent neutralizing monoclonal antibodies.

## 4 Methods

### 4.1 Antibodies

The mouse anti-E2 AP33 has been described previously (Owsianka et al., 2005). The anti-E1 A4 (Dubuisson et al., 1994) mAbs was generously provided by J. Dubuisson. The human anti-E2 mAbs CBH-2, CBH-4B, CBH-5, CBH-7 and HC-11 (Hadlock et al., 2000, Keck et al., 2004) have been a kind gift from S. K. Foung. Anti-Flag M2 mAb was purchased from Sigma-Aldrich.

### 4.2 Cell lines

Human epithelial kidney (HEK) 293T cells and human hepatoma Huh-7 and Huh7-J20 (Iro et al., 2009) cells were propagated in Dulbecco’s modified essential medium (DMEM) supplemented with 10% fetal bovine serum, in standard cell culture conditions.

### 4.3 Construction of E1E2-Flag expression plasmids

The plasmid encoding HCV genotype 1a strain H77c (Choo et al., 1989) E1E2 (GenBank accession number AF011751) was previously described by (Yanagi et al., 1997), while the two patient-derived clones – UKN 1b.12.6 (AY734975) and UKN 2a.2.4 (AY734979) (Lavillette et al., 2005), were obtained from the laboratory of J. Ball, University of Nottingham, UK. For genotypes 1a and 1b, the Flag sequence was fused in-frame to the E2 sequence by classic insertion PCR. In brief, using the H77 and UKN1b as templates, a PCR was performed using the following primers: 1a_fwd 5′-GGCGCCAAGGACTACAAGGACGATGACGATAAACAGAACATCCAACTGATC-3′ and 1a_rev 5′-CGTATCATTTGCACCCCAGCTGTAGGTAGGCGCGCCCGACCTGTC-3′; and 1b_fwd 5′-CTTTAGATCTGGACCGTCTGACTACAAGGACGATGACGATAAACAGAAGATCCAACTTGTGAA-3′ and 1b_rev 5′-CATCCATGTACAGCCGAACC-3′. The E1E2 vector and the PCR products were cut with EheI-*Asc*I and *Bgl*II-Bsp1407l, respectively, and ligated.

The cloning strategy for gt2a was based on a three-step assembly PCR, due to its characteristic sequence. In the first step, using primers 2a_1fwd 5′-GGAGCGTGGGCTAAGGTCATC-3′ and 2a_1rev 5′-TACAAGTCACATCCACGCTCTCTGATGTTCCTGCTACTGCTATTT-3′ and the UKN 2a2.4 as a matrix, a 355 bp product (PCR1) was amplified, which had a *Sma*I restriction site near its 5′ end and the Flag sequence at its 3′ end. Then using primers 2a_2fwd 5′-GACTACAAGGACGATGACGATAAACAAAACATCCAGCTTATCAAC-3′ and 2a_2rev 5′-TCGTGTGCCGGCGGTGTTCCC-3′, a 492 bp product was obtained, containing Flag at its 5′ and *Xcm*I restriction site near its 3′ end (PCR2). Finally, PCR1 and PCR2 were assembled into an 823 bp product using primers 2a_1fwd and 2a_2rev (PCR3). The PCR3 fragment was digested with SmaI and XcmI restriction enzymes, ligated to UKN 2a2.4 and sequenced. The sequencing confirmed presence of Flag and absence of any additional mutations. The new constructs were further referred to as E1E2-Flag.

### 4.4 Expression and purification of E1E2-Flag

293T cells were transfected with the E1E2-Flag plasmids using the CaPO_4_ method. 48hrs after transfection the cells were lysed using the mild buffer and protein expression was analyzed via Western blotting. The binding was performed in a batch format – 1 ml of cell lysate was added to 50 µl washed resin. For the negative control, 1 ml of lysate from cells transfected with GFP was used. All samples and controls were agitated gently on a roller shaker for 2 h at RT. All unbound protein was removed by washing the resin three times with 1 ml TBS. After the final wash, all the supernatant was removed and the bound Flag-tagged protein eluted from the agarose by incubation with 0.5 ml of 0.1 M glycine-HCl, pH 3.5 buffer. The test tubes were incubated for 5 min at room temperature with gentle shaking. The agarose was not left in this low pH buffer for more than 10 min in order not to dissociate the M2 mAb. The probes were centrifuged for 30 s at 7000 × g and the supernatants were immediately transferred to fresh Eppendorf test tubes containing 10 µl of 1 M Tris HCl, pH 8.8. For immediate use the supernatants were stored on ice or alternatively frozen at −20 °C for long-term storage.

### 4.5 *In situ* immunocytochemical staining

E1E2 transfected 293T cells monolayers were washed with PBS, frozen at 70 °C for 15 min, and fixed with 4% paraformaldehyde. Cells were probed with anti-E2 mAb AP33 diluted 1:1000 in PBS with 5% fetal bovine serum (FBS), 0.1% sodium azide, 1% Tween-80 or anti-Flag M2 mAb diluted 1:500. Secondary HRP-conjugated anti-mouse antibody (Santa Cruz) diluted 1:2000 in PBS with 5% FBS, 1% Tween-80 was used. The reaction was developed with the Nova-RED substrate kit (Vector Laboratories).

### 4.6 GNA-capture ELISA

Detection of E1E2 by ELISA was performed as described by (Tarr et al., 2007). Briefly, 96-well plates coated with GNA lectin were used to capture glycoproteins from lysates of HEK 293T cells transiently expressing wt or mutated E1E2, prepared as described above. Lysate from mock-transfected cells served as a negative control. Bound proteins were detected by using appropriate antibodies followed by anti-species IgG–HRP conjugate and TMB substrate (Sigma-Aldrich).

### 4.7 E1E2 co-immunoprecipitation

293T cells, transiently expressing E1E2 wt or E1E2-Flag, were metabolically radiolabeled with [^35^S]-Met and lysed 48 h post transfection, proteins were immunoprecipitated with anti-E2 AP33 mAb, separated on 12% SDS-PAGE (reducing and non-reducing) and detected by radiography.

### 4.8 CD81-binding assay

CD81 binding was performed as previously described by (Tarr et al., 2007). In brief, Glutathione Sepharose beads were first coated with recombinant GST-hCD81-LEL for 2 h at 4 °C and washed twice in PBS–1% Triton X-100. They were then divided into aliquot parts and incubated overnight with lysates from HEK 293T cells expressing HCV E1E2 wt or Flagged, as well as with mock control. The samples were washed 4x in PBS–1% Triton X-100 and once in water before addition of Laemmli buffer and resolved on 12% SDS-PAGE gel. As a negative control, GST bound to glutathione Sepharose was used. E2 was detected with AP33 mAb.

### 4.9 Animals and vaccinations

Animal experiments were approved by local animal ethics committees and were performed in accordance with national and international laws and policies. Groups of six female 5–6 weeks of age C57BL/6 mice were vaccinated intraperitoneally with E1E2-Flag (5 μg per mouse). Recombinant protein was formulated by being mixed 1:1 with incomplete Freund’s adjuvant (IFA) immediately before immunization. Blood samples were collected by tail-bleeding and total blood by cardiac puncture. All in vivo procedures were performed under deep anesthesia.

### 4.10 HCVcc neutralization

Antibody inhibition assays were performed using Huh7-J20 cells, and virus infectivity levels were determined by secreted alkaline phosphatase (SEAP) reporter assay, as described previously (Iro et al., 2009). Briefly, Huh7-J20 cells were plated out at a density of 3 × 10^3^ per well in a 96-well plate. ∼50 FFU of virus was pre-incubated at 37 °C for 1 h with the appropriate inhibitory or control antibody prior to infecting cells. At 3 h post infection, the inoculum was replaced with fresh medium and incubated for 48 h. The infectivity was determined by measurement of the SEAP activity released into the medium.

In this assay, H77/JFH1/HQL (HCV1a) and JFH1-AM71 (HCV2a) viruses were used. The adaptive mutations in HQL, (Y835H, K1402Q and V2440L) and AM71, increased the viral titers up to 1 × 10^6^ CCID_50_/ml (data not shown). The analyzed sera were diluted 1:50, the control anti-E2 AP33 antibody was used in inhibitory concentration of 100 μg/ml. Controls included non-infected cells and cells infected with mock treated viruses (no antibodies).

### 4.11 Statistical analysis

Statistical analysis and graphs were made using Prism 7 for OS X software (GraphPad). A 2-tailed *t*-test (paired or unpaired as appropriate) was used to determine if there were significant differences.
